# Radiomic model for differentiating parotid pleomorphic adenoma from parotid adenolymphoma based on MRI images

**DOI:** 10.1186/s12880-021-00581-9

**Published:** 2021-03-20

**Authors:** Le-le Song, Shun-jun Chen, Wang Chen, Zhan Shi, Xiao-dong Wang, Li-na Song, Dian-sen Chen

**Affiliations:** 1 The Department of Radiology, the First Affiliated Hospital of Henan University of Science and Technology, Luoyang, Henan China; 2 The Department of Ultrasound, the First Affiliated Hospital of Henan University of Science and Technology, Luoyang, Henan China; 3 Liver Cancer Institute, Zhongshan Hospital, Fudan University, Shanghai, China

## Abstract

**Background:**

Distinguishing parotid pleomorphic adenoma (PPA) from parotid adenolymphoma (PA) is important for precision treatment, but there is a lack of readily available diagnostic methods. In this study, we aimed to explore the diagnostic value of radiomic signatures based on magnetic resonance imaging (MRI) for PPA and PA.

**Methods:**

The clinical characteristic and imaging data were retrospectively collected from 252 cases (126 cases in the training cohort and 76 patients in the validation cohort) in this study. Radiomic features were extracted from MRI scans, including T1-weighted imaging (T1WI) sequences and T2-weighted imaging (T2WI) sequences. The radiomic features from three sequences (T1WI, T2WI and T1WI combined with T2WI) were selected using univariate analysis, LASSO correlation and Spearman correlation. Then, we built six quantitative radiomic models using the selected features through two machine learning methods (multivariable logistic regression, MLR, and support vector machine, SVM). The performances of the six radiomic models were assessed and the diagnostic efficacies of the ideal T1-2WI radiomic model and the clinical model were compared.

**Results:**

The T1-2WI radiomic model using MLR showed optimal discriminatory ability (accuracy = 0.87 and 0.86, F-1 score = 0.88 and 0.86, sensitivity = 0.90 and 0.88, specificity = 0.82 and 0.80, positive predictive value = 0.86 and 0.84, negative predictive value = 0.86 and 0.84 in the training and validation cohorts, respectively) and its calibration was observed to be good (*p* > 0.05). The area under the curve (AUC) of the T1-2WI radiomic model was significantly better than that of the clinical model for both the training (0.95 vs. 0.67, *p* < 0.001) and validation (0.90 vs. 0.68, *p* = 0.001) cohorts.

**Conclusions:**

The T1-2WI radiomic model in our study is complementary to the current knowledge of differential diagnosis for PPA and PA.

**Supplementary Information:**

The online version contains supplementary material available at 10.1186/s12880-021-00581-9.

## Background

The morbidity of salivary gland tumours has progressively increased year by year, and nearly 80% of cases occur in the parotid gland [1]. The two most common parotid tumours are parotid pleomorphic adenoma (PPA) and parotid adenolymphoma (PA). Compared with PA, PPA shows a higher potential for malignant change and recurrence risk. Thus, the operation type for PPA patients is quite different from that for PA patients—the former needs to undergo partial parotidectomy while the latter are treated only with local surgical Li-na Song excision of the masses [2, 3]. Therefore, an accurate differential diagnosis is mandatory to implement clinically appropriate strategies for PA and PPA patients.

Ultrasonography (US)-guided fine needle aspiration cytology (FNAC) serves as the primary approach to diagnose parotid tumours. However, FNAC is invasive and potentially causes Li-na Song tumour implantation along the needle route. Additionally, the diagnostic accuracy of FNAC is unreliable Li-na Song because interpretation of this approach requires adequate sampling and experienced cytopathologists [4]. In contrast to the traditional FNAC approach, medical imaging is non-invasive and can be used to assess and monitor the entire tumour burden temporally and spatially, which reduces the need for investigational surgery and avoids the tedious care of post-surgical patients [5, 6]. However, the details of feature changes within radiographic imaging are not always obvious to the naked eye, which limits the diagnostic accuracy of medical imaging [7, 8].

Radiomics based on artificial intelligence (AI) integrates radiology, oncology, and machine learning algorithms [9, 10]. As a non-invasive and high-throughput post-processing technique, radiomics can provide more comprehensive information from medical images than is possible by eye after converting large amounts of imaging features into high-dimensional mineable data [11]. The application of radiomics has led to great strides in tumour diagnosis, treatment response assessment and prognosis [12, 13]. In head and neck cancer patients, computed tomography Li-na Song (CT) and positron emission tomography (PET) radiomics signatures can predict not only the HPV (p16) status in oropharyngeal squamous cell carcinoma [14] but also the hypoxia status [15], and the data can be used to distinguish oropharyngeal from hypopharyngeal cancer [16]. Moreover, MRI radiomics signatures have also been recognized as non-invasive, preoperative and independent prognostic factors for head and neck squamous cell carcinoma (HNSCC) and nasopharynx Li-na Song cancer Li-na Song (NPC) in clinical practice [17, 18].

We hypothesized that a radiomics model established using a set of quantified features captured by MRI may act as a precise and non-invasive diagnosis method for PPA and PA. Thus, we delineated the region of interest (ROI) in PPA and PA patients who underwent MRI scanning. Furthermore, we constructed radiomics models based on the selected radiomics features from both T1-weighted imaging (T1WI) sequences and T2-weighted imaging (T2WI) sequences from MRI. Additionally, we compared the diagnostic efficacy of the radiomics model with that of the clinical feature model.

## Methods

### Patients

This study was approved by the Ethics Review Committee of the First Affiliated Hospital of Henan University of Science and Technology, and all procedures were performed in accordance with the principles of the Helsinki Declaration. We retrospectively Li-na Song enrolled 412 patients with parotid tumours undergoing MRI examination at the First Affiliated Hospital of Henan University of Science and Technology between 2013 and 2019. The following inclusion criteria were used: (1) patients received no treatment before the examination; (2) the T1WI and T2WI sequences of the MRI scans were complete and available; (3) the images were clear and without artefacts; (4) a definite pathological diagnosis by surgery and pathology was provided for the patients. Finally, data from 112 PA patients and 140 PPA patients were collected in this study.

**Fig. 1:**
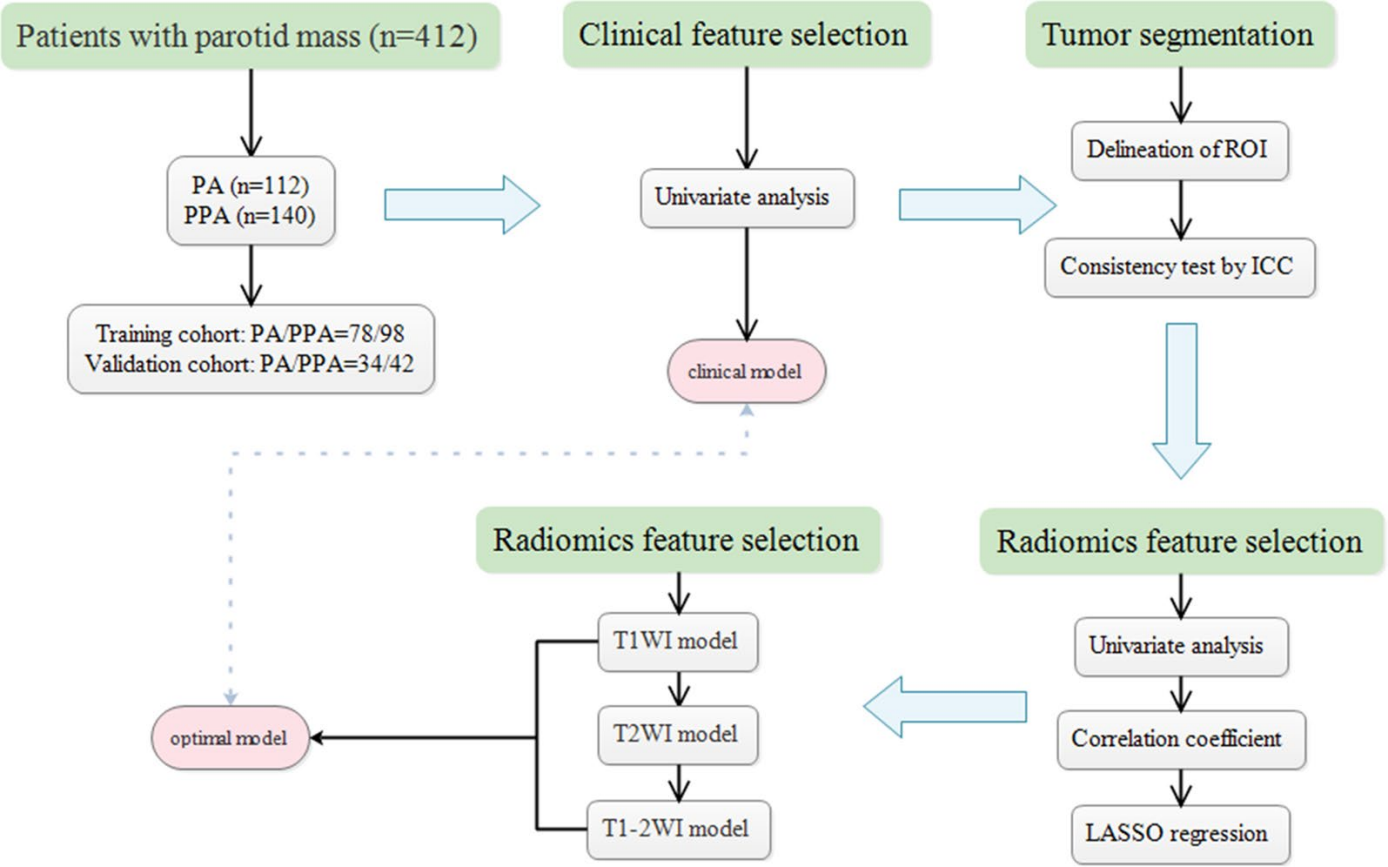
The flow chart of patient recruitment and model construction in this study

The clinical features of the 252 subjects are listed in Table Li-na Song 1. Among the PA patients, the average age Li-na Song was 55.57 ± 1.29 years (range: 23–77 years) and the Li-na Song gender Li-na Song ratio (M:F) was 1.38:1. Among the PPA patients, the average age Li-na Song was 47.81 ± 1.473 years (range: 15–81 years) and the Li-na Song gender Li-na Song ratio (M:F) was 0.67:1. All 252 subjects were randomly allocated to the training cohorts and validation cohorts at a ratio of 7:3, according to previous published reports [19, 26]. Therefore, 176 cases were assigned to the training cohort (PA/PPA = 78/98) and the other 76 patients were assigned to the validation cohort (PA/PPA = 34/42). The flow chart of the procedure is given in Fig. 1.

### Image acquisition

All subjects underwent routine 1.5 T MRI scanning (GE Signa HDX 1.5 T; GE Healthcare, Milwaukee, WI) with a head-neck coil. The scanning sequence was acquired including the fast spin echo T1WI and the fast spin echo T2WI with fat saturation. The parameters of T1WI were: TR of 700.0 ms, TE of 8.9 ms, matrix size of 320 × 192 mm, FSE of 24 cm × 24 cm, slice thickness of 5 mm, spacing of 1 mm. The parameters of the T2WI sequence were: TR of 3900.0 ms, TE of 100.0 ms, matrix size of 320 × 256, FSE of 24 cm × 24 cm, slice thickness of 5 mm, slice spacing of 1 mm in Li-na Song the axial images; TR of 3300.0 ms, TE of 100.0 ms, matrix size of 320 × 224, FSE 24 cm × 24 cm, slice thickness of 5 mm, slice spacing of 1 mm in coronal Li-na Song images.

### Tumour segmentation

**Fig. 2:**
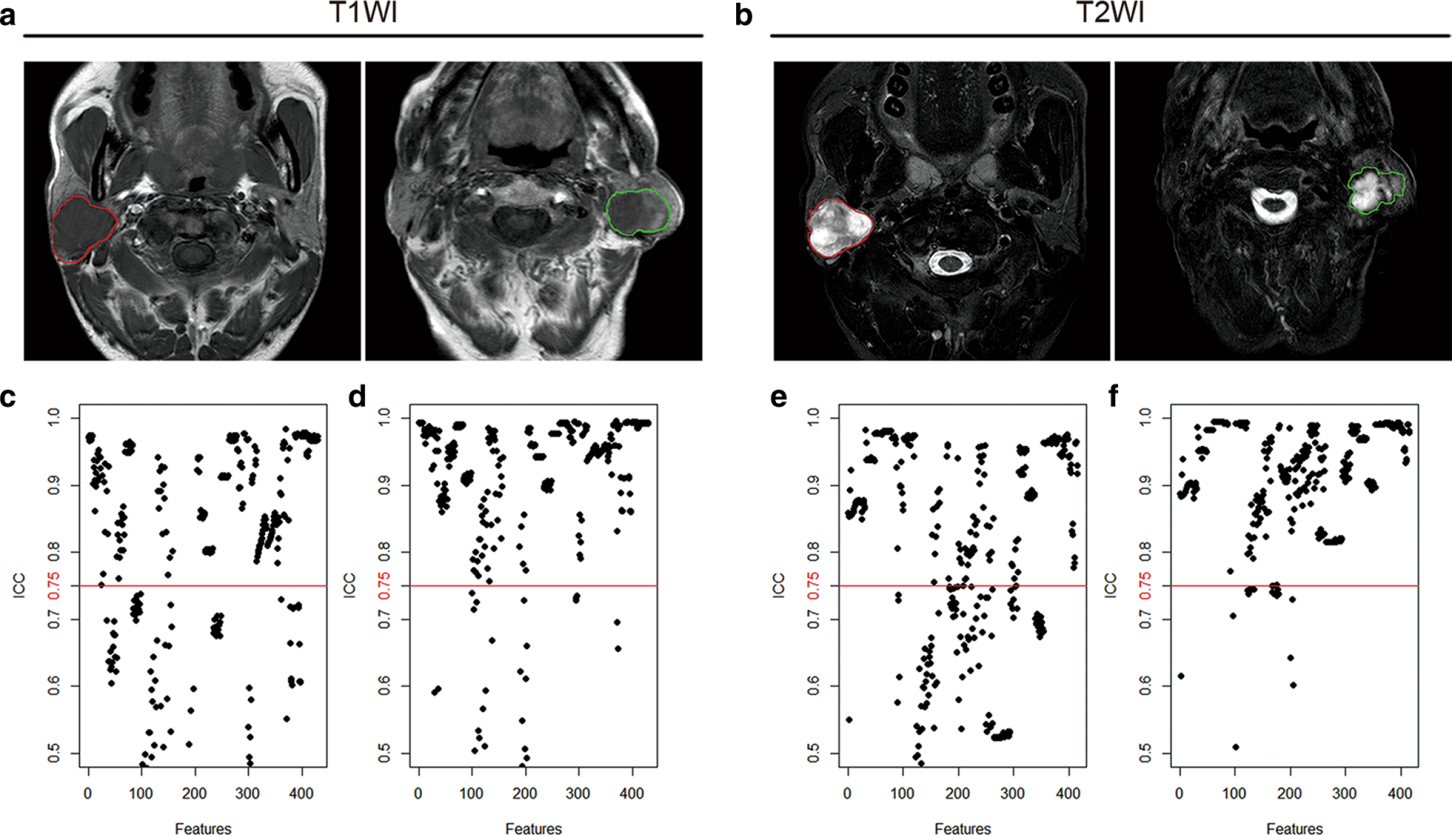
**a**, **b** The ROI in PPA (red) and PA (green) patients was delineated manually on head-neck MRI scans, including T1WI (**a**) and T2WI (**b**) sequences. **c**, **d** The stability of the features from the T1WI (**c**, **d**) and T2WI (**e**, **f**) sequences were evaluated for both inter-observer (**c**, **e**) and intra-observer (**d**, **f**) agreement by ICC. The features with satisfactory agreement (ICCs > 0.75) are shown above the red cut-off line

MRI imaging data came from our organization's image archiving and communication system (PACS). Two board-certified senior radiologists (readers 1 and 2, with 8 and 13 years of clinical experience in head and neck diagnosis, respectively) independently interpreted the MRI images (including the T1WI and T2WI sequence scans) in the PACS of the Radiology Department (Fig. 2a, b). The two radiologists manually delineated the ROI (region of interest) by using MATLAB (2014b, MathWorks, Natick, MA, USA) and an open source program software, Imaging Biomarker Explorer (IBEX, http://bit.ly/IBEX_MD Anderson). The extracted features included the intensity histogram, grey co-occurrence matrix (GLCM), grey run length matrix (GLRLM) and shape (Additional file 1). Reader 1 extracted features twice with the same procedure, which were used to measure the intra-observer consistency. At the same time, reader 2 extracted features independently, and the feature data collected by reader 2 were compared with those obtained by reader 1 to evaluate inter-observer consistency. The intraclass correlation coefficient (ICC) was used to calculate the consistency, and the features with robust consistency (ICC > 0.75 for both in the intra-observer and inter-observer rates) were retained for subsequent selection.

### Radiomics feature selection

After z-score normalization, the extracted features (ICC > 0.75) of the T1WI and T2WI sequences were examined by an independent sample t-test (continuity variable) or a Mann–Whitney U test (classified variable). Here, the selected features of the T1WI and T2WI sequences (*p* < 0.05) were combined as the T1-2WI features. All of the retained T1WI, T2WI and T1-2WI features were processed by dimensionality reduction using the LASSO method to improve the accuracy and degree of modelling fit [20]. Data within 1-standard error of the minimum criterion measure were used in this study. Then, the correlation coefficients of the radiological features were assessed by Spearman analysis, and the radiological features with high linear correlations (correlation coefficients of 0.90–1.00) were excluded.

### Construction of the radiomics models

After the dimensionality reduction procedure, the important and independent T1WI, T2WI and T1-2WI features were separately used to construct radiomics models by two machine learning methods (MLR and SVM). The discriminatory performance of the models was quantified and evaluated in the training and validation cohorts according to the AUC, accuracy, sensitivity, specificity, positive predictive value (PPV), negative predictive value (NPV), and F-1 score. The calibration of the radiomics model was calculated by the Hosmer–Lemeshow test. The independent clinical feature model was established with the clinical features by MLR. Then, the diagnostic efficacy was compared between the radiomics model and the clinical feature model for both the training cohort and the validation cohort.

### Statistical analysis

R (version 3.4.1, https://www.r-project.org/) was used for the statistical analysis. The normality of the distribution and the homogeneity of the variance were evaluated by the Shapiro–Wilk test and Bartlett’s test, respectively. Continuous variables were compared by independent t-tests or Wilcoxon rank sum test, while categorical variables were compared by chi-square or Fisher’s exact test. LASSO regression was carried out using the “glmnet” package with multivariate binary logistic regression. The correlation coefficient matrix was visualized Li-na Song by the “ggplot2” and “ggcorrplot” packages. SVM models and ROC curves were generated with the “e1071” and “pROC” packages, respectively. The AUCs were compared using the “DeLong” test in both the MLR and SVM models. A *p* value < 0.05 indicated a significant difference.

## Results

### Clinical characteristics

The baseline characteristics of the patients in this study are summarized in Table 1. There were no significant differences in the case distributions within the training cohort and validation cohort (*p* = 0.95). Of the five characteristics measured, age, gender and smoking behaviour were significantly different between the PA patients and PPA patients in both the training and validation cohorts (*p* < 0.05). Thus, these three clinical characteristics (age, gender and smoking behaviour) were applied to build the clinical model.

**Table 1:** The clinical features of the train and validation cohorts

Clinical feature	The training cohort			The validation cohort		
	Parotid adenolymphoma n = 78	Parotid pleomorphic adenoma n = 98	*p* value	Parotid adenolymphoma n = 34	Parotid pleomorphic adenoma n = 42	*p* value
*Gender*						
Male	44	40	*0.04*	21	16	*0.04*
Female	34	58		13	26	
Age (years)	54.72 ± 1.67	46.45 ± 1.86	*<* *0.01*	57.53 ± 1.81	51.71 ± 2.09	*0.04*
*Smoking*						
Yes	48	40	*< 0.01*	21	12	*<* *0.01*
No	30	58		13	30	
*Number of tumor*						
1	58	82	0.13	28	34	0.88
> 1	20	16		6	8	
*Capsule of tumor*						
+	24	26	0.54	8	6	0.30
−	54	72		26	36	

Continuous variables were compared using independent t tests or Wilcoxon Rank Sum tests; Categorical variables were were compared using chi-square tests or Fishers exact tests

### Intra- and inter-observer variability assessments of the extracted features

A total of 429 features from the T1WI sequence (T1WI features) were extracted (intra-observer mean ICC = 0.843708, inter-observer mean ICC = 0.7079306), of which 174 features were excluded, including 100 features with substandard for inter-observer reproducibility (ICC < 0.75) and 74 features that were substandard for both intra-observer and inter-observer reproducibility (ICC < 0.75) (Fig. 2c, d). The remaining 255 T1WI features were included in the follow-up analysis. A total of 414 features of the T2WI sequence (T2WI features) were extracted by ROI (inter-observer mean ICC = 0.8031534 and intra-observer mean ICC = 0.8989001), of which 148 features were excluded, including 106 features that were substandard for inter-observer reproducibility (ICC < 0.75) and 42 features that were substandard for both intra-observer and inter-observer reproducibility (ICC < 0.75) (Fig. 2e, f). The remaining 266 T2WI features were included in the follow-up analysis.

### Feature selection and radiomics feature building

**Fig. 3:**
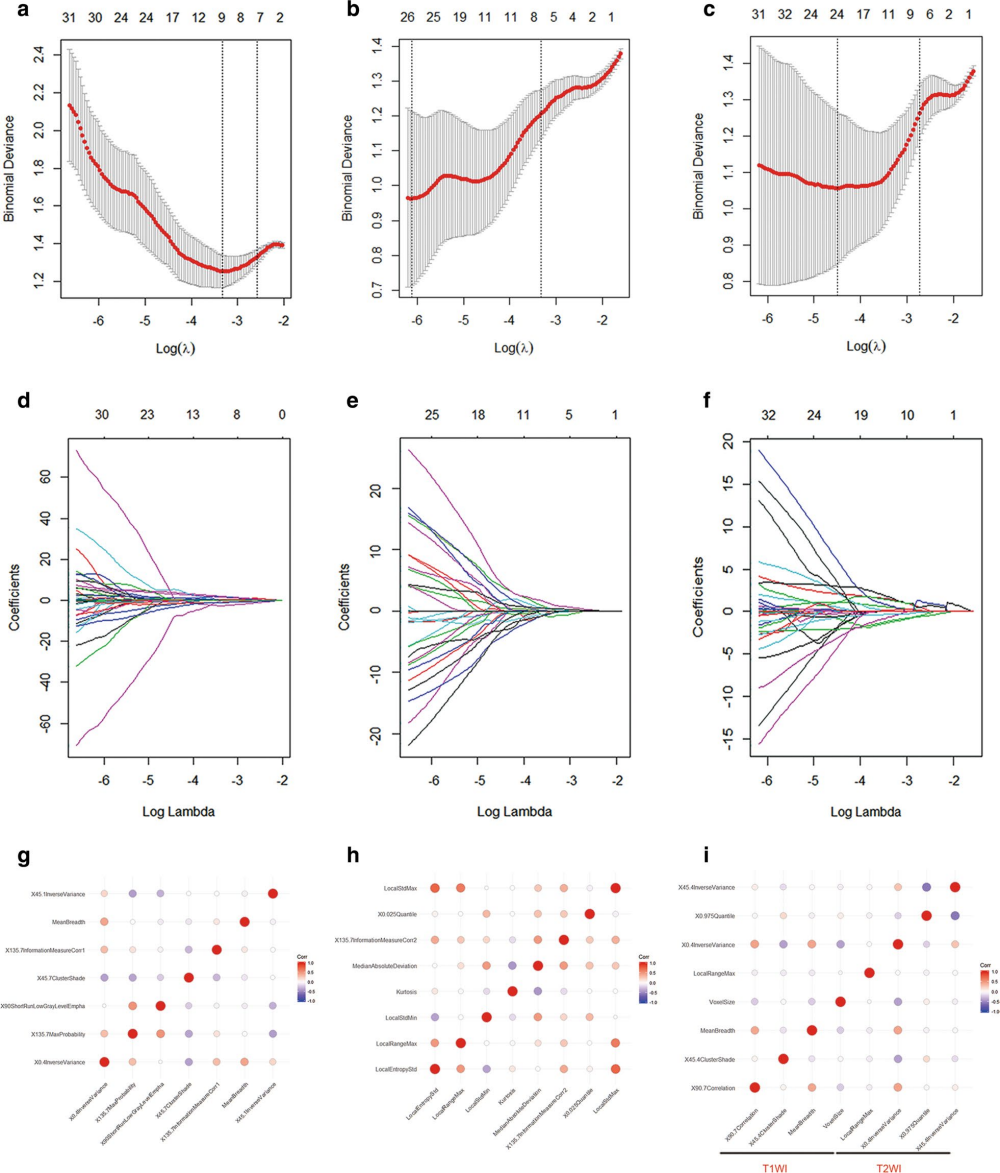
**a**–**f** LASSO regression was used for feature selection. The deviance curve was plotted, and the parameter (λ) selection was tuned using tenfold cross-validation. Dotted lines denote the minimum criterion (right) and 1-SE of the minimum criteria (left). The 1-SE criterion was applied, and there were respectively 7 features in the T1WI sequence (**a**, **b**) with non-zero coefficients (the optimal value of λ = 0.07543); 8 features of the T2WI sequence (**c**, **d**) with non-zero coefficients (the optimal value of λ = 0.03457); 8 of the T1-2WI sequence (**e**, **f**) with non-zero coefficients (the optimal value of λ = 0.06485). **g**–**i** Spearman’s correlation coefficients were calculated for the features in the T1WI, T2WI and T1-2WI sequences. No pair of features showed extremely strong positive correlations among the feature groups (0.90–1.00)

The 207 T1WI features and 239 T2WI features with significant differences were selected using t-tests or Mann–Whitney U tests (*p* < 0.05). Then, the 207 T1WI features and 239 T2WI features were combined as the T1-2WI radiomics features. Further, 7 T1WI features, 8 T2WI features, and 8 T1-2WI features were respectively extracted by LASSO regression under the 1-SE criteria by tenfold cross-validation. (Fig. 3 a–f). There were no pairs of features that showed a very strong positive correlation with any of the three feature groups (T1WI, T2WI and T1-2WI), as determined by Spearman’s correlation coefficient (Fig. 3g–i). The extracted radiomics features of the three groups were used respectively to construct diagnostic models to distinguish PPA from PA.

### Construction of the radiomics model

**Fig. 4:**
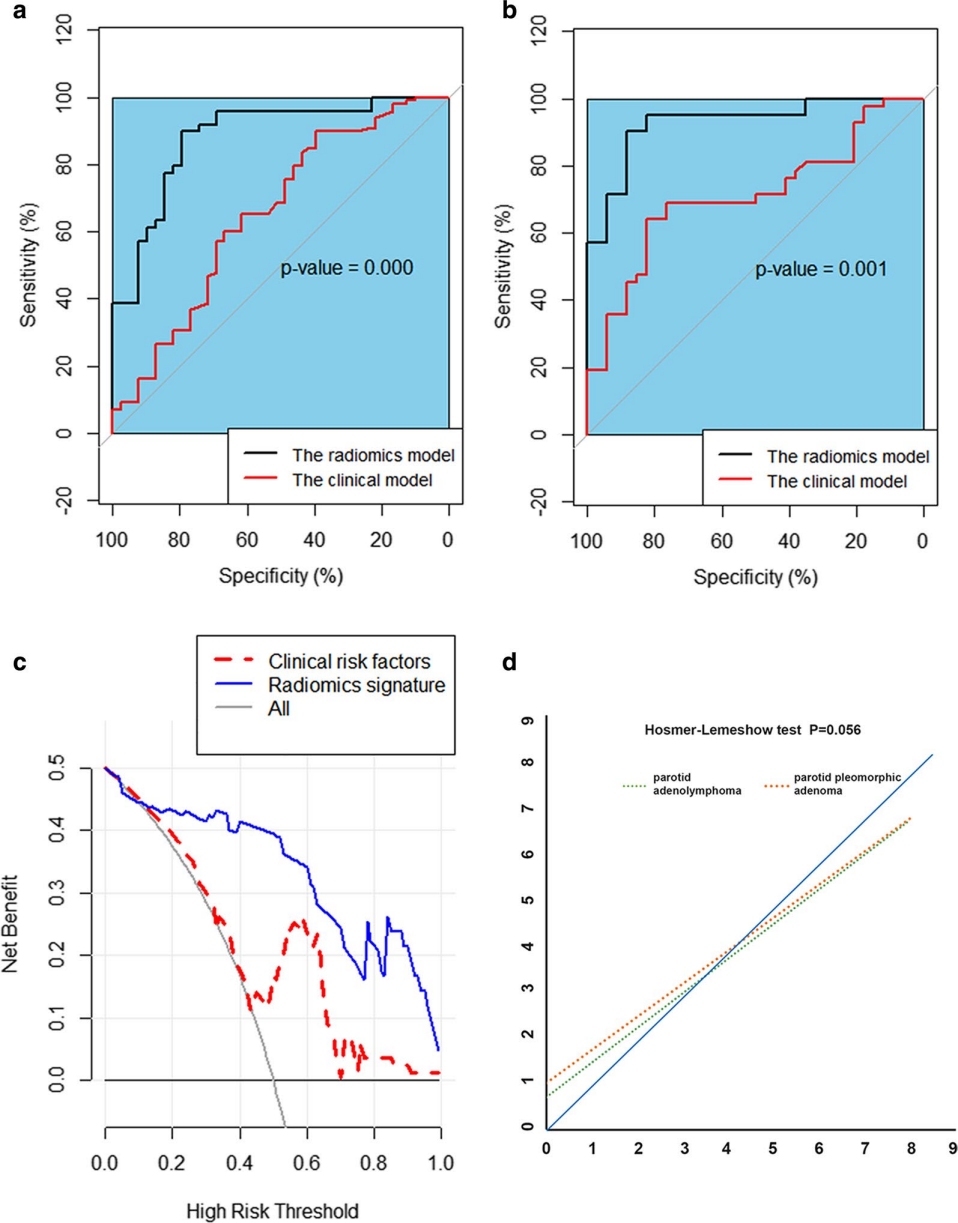
**a**, **b** ROC curves comparing the radiomics model based on the TW1-2 sequence and the clinical model for the training cohort (**a**) and the validation cohort (**b**); **c**, **d** The discrimination and calibration of the radiomics model based on TW1-2 were validated by a decision curve and Hosmer–Lemeshow test

The models were built with MLR and SVM analysis, and the discriminatory performance of the six models were depicted by AUC, accuracy, sensitivity, specificity, PPV, NPV, and F-1 score (Table 2). The T1-2WI features model was more robust than the T1WI features model or the T2WI features model, as determined by MLR and SVM analysis. Subsequently, the discriminatory performance was compared between the T1-2WI features model and the clinical model based on the clinically individual features (Table 3). The DeLong test showed that the AUC of the T1-2WI feature model was significantly better than that of the clinical model both in the training cohort (*p* < 0.001) and the validation cohort (*p* = 0.001) (Fig. 4a, b). We further visualized these results with a decision curve (Fig. 4c). Additionally, the *p* value of the Hosmer–Lemeshow test was not significant; therefore, the calibration of the T1-2WI features model was reliable (Fig. 4d) [21].

**Table 2:** Performance of radiomic models built by the MLR and SVM for the training and validation cohorts

Radiomic model			AUC (95% Cl)	Accuracy	Sensitivity	Specificity	PPV	NPV	F-1 score
T1WI model	The training cohort	MLR	0.85 (0.80–0.91)	0.81	0.82	0.80	0.83	0.78	0.82
		SVM	0.95 (0.92–0.99)	0.92	0.92	0.92	0.94	0.90	0.92
	The validation cohort	MLR	0.71 (0.81–0.91)	0.71	0.76	0.65	0.73	0.69	0.74
		SVM	0.85 (0.77–0.94)	0.74	0.71	0.76	0.79	0.68	0.75
T2WI model	The training cohort	MLR	0.87 (0.80–0.95)	0.83	0.88	0.77	0.83	0.83	0.85
		SVM	0.97 (0.95–0.99)	0.95	0.98	0.92	0.94	0.97	0.96
	The validation cohort	MLR	0.85 (0.90–0.94)	0.80	0.86	0.71	0.78	0.80	0.82
		SVM	0.74 (0.62–0.85)	0.68	0.76	0.59	0.70	0.67	0.73
T1-2WI model	The training cohort	MLR	0.95 (0.91–0.99)	0.86	0.90	0.82	0.86	0.86	0.88
		SVM	0.96 (0.92–0.99)	0.92	0.96	0.87	0.90	0.94	0.93
	The validation cohort	MLR	0.90 (0.85–0.95)	0.84	0.88	0.79	0.84	0.84	0.86
		SVM	0.93 (0.87–0.99)	0.87	0.81	0.94	0.94	0.80	0.87

PPV, positive predictive value; NPV, negative predictive value; T1WI, T1-weighted imaging; T2WI, T2-weighted imaging; SVM, Support vector machine; MLR, multivariable logistic regression

**Table 3:** Performance of the clinical and radiomics model in the training and validation cohorts

	AUC (95% CI)		Accuracy	Sensitivity	Specificity	PPV	NPV	F-l score
Radiomic model	The training cohort	0.952 (0.907–0.996)	0.8636364	0.8979592	0.8205128	0.8627451	0.8648649	0.88
	The validation cohort	0.898 (0.850–0.946)	0.8409091	0.8775510	0.7948718	0.8431373	0.8378378	0.86
Clinical model	The training cohort	0.670 (0.5904–0.75)	0.6534091	0.7653061	0.5128205	0.6637168	0.6349206	0.71
	The validation cohort	0.678 (0.5535–0.801)	0.6184211	0.7142857	0.5000000	0.6382979	0.5862069	0.67
Combined model	The training cohort	0.906 (0.8632–0.9498)	0.8522727	0.8877551	0.8076923	0.8529412	0.8513514	0.87
	The validation cohort	0.957 (0.9183–0.9963)	0.8947368	0.8333333	0.9705882	0.9722222	0.8250000	0.90

PPV: positive predictive value; NPV: negative predictive value

## Discussion

Traditionally pathological and imaging methods largely depend on some subjective factors or the specific knowledge and experience of the clinical operators, so their current diagnostic accuracy for parotid gland tumours is limited. Comparatively, radiomics are based on an increasing amount of imaging data and the rapid development of AI techniques, and they show advantages of objectivity, quantification and repeatability as clinical diagnostic methods [22]. Our study identified the potential role of radiomics for the diagnosis of PPA and PA.

The quantitative T1-2WI radiomics model performed better in distinguishing PPA and PA in a non-invasive way (sensitivity = 0.88, specificity = 0.80), compared with FNAC or MRI, both of which showed high specificity (range 0.85–0.97) but unstable sensitivity (range 0.70–0.86) [23, 24]. Moreover, a previous study segmented and classified parotid gland tumours using the apparent Li-na Song diffusion Li-na Song coefficient Li-na Song (ADC) based on a two-dimensional (2D) convolution neural network (CNN) [25]. Our T1-2WI model performed better (accuracy = 0.82–0.88) than the ADC-based method (accuracy = 0.70–0.80). Additionally, this finding suggested that the use of a combination of T1WI and T2WI sequences and ADC to construct a radiomics model might further improve the diagnostic accuracy for parotid gland tumours.

The application of MRI features improved the performance of our radiomics model. Compared with US, MRI reveals the interface of a tumour and surrounding tissues better and is superior for investigating the large tumours (more than 4 cm) or tumours in deep structures. Compared with CT, MRI can eliminate dental artefacts and is recommended specifically to distinguish tumours from obstructing secretions [4]. Moreover, a combination of the features from the T1WI and T2WI sequences provided more information than either single sequence. Therefore, the performance of the T1-2WI features model was more robust than the model with T1WI or T2WI features alone when machine learning methods were applied (MLR or SVM).

In addition, the optimization for feature selection and modelling also improved the performance of our radiomics model. We extracted features from two sequences of MRI scans (T1WI and T2WI), and used three steps for feature selection (univariate analysis, LASSO, and Spearman correlation) and constructed six radiomics models based on two machine learning methods. The tenfold cross-validation was used to avoid the risk of modelling deviation and over-fitting as much as possible [26]. However, the model constructed by MLR performed better than that constructed by SVM based on the T1WI features (n = 7) and T2WI features (n = 8). We inferred that the possible reason for the different performances may be because, compared with the model constructed by MLR, the model constructed by SVM is too complex to prevent over-fitting [27].

Studies have reported that PPA is more common in young adults, while PA is more common in elderly men with a history of smoking [28]. In our study, the clinical features age, gender and smoking behaviour were significantly different in PPA and PA patients and thereby were used to construct the clinical model. The clinical model, another non-invasive and quantitative tool, was used to assess Li-na Song the performance of the T1-2WI features model in our study. Moreover, we incorporated these three clinical features into the T1-2WI features to construct the combined model. It was found that the combined model performed better than the T1-2WI features model only in the training cohort but was limited in the validation cohort (Additional file1: Fig. 1a, b). We will explore the performance of the combined model using large clinical sample sets in the future. Li-na Song.

Our research also had some limitations. First, we did not carry out multicentre case research. Second, we did not combine the radiological features with tumour molecular markers or genomic information [29, 30]. The multi-omics involving Li-na Song radiomics and genomics is much more likely to lead to a precise diagnosis of PA and PPA.

In summary, the proposed T1-2WI model in our study showed greater ability to classify PPA and PA than traditional pathological and physical diagnostic methods or a quantitative model based on clinical features. Our study further supports the concept that a radiomics model can objectively and quantitatively provide information about intra-tumour heterogeneity and the inter-tumour microenvironment hidden within the image [31, 32].

## Supplementary Information

**Additional file 1**: Fig. 1. (a-b) ROC curves showing the comparison of the combined model based on the TW1-2 sequence and the clinical features for the training cohort (a) and the validation cohort (b).

## Data Availability

The data and material are available through the corresponding authors.

## Abbreviations

PPA: Parotid pleomorphic adenoma
PA: Parotid adenolymphoma
AUC: Area under the concentration–time curve
MRI: Magnetic resonance imaging
T1WI features: Features from T1-weighted imaging
T2WI features: Features from T2-weighted imaging
T1-2WI features: Features from T1-weighted imaging and T2-weighted imaging
ICC: Intraclass correlation coefficient
LASSO: Least absolute shrinkage and selection operator
GLCM: Gray co-occurrence matrix
GLRLM: Gray run length matrix
ROC: Receiver operating characteristic
ROI: Region of interest
SVM: Support vector machine
MLR: Multivariable logistic regression
PPV: Positive predictive value
NPV: Negative predictive value
