# Supplementary material for: Radiomic model for differentiating parotid pleomorphic adenoma from parotid adenolymphoma based on MRI images
Source: BMC Med Imaging. 2021 Mar 20;21:54. doi: 10.1186/s12880-021-00581-9 (PMC7981906; doi:10.1186/s12880-021-00581-9)
Supplement: Supplementary file 1 — Additional file 1: Fig. 1. (a-b) ROC curves showing the comparison of the combined model based on the TW1-2 sequence and the clinical features for the training cohort (a) and the validation cohort (b). [file 12880_2021_581_MOESM1_ESM.pdf]

Additional file 1

Category: Intensity Histogram

Variable	Description	Parameters
Inter Quartile Range	The interquartile range of the occurence probability values in the histogram.	N/A
Kurtosis	Measure the peakedness of the occurence probability values in the histogram.	N/A
Mean Absolute Deviation	The mean absolute deviation of the occurence probability values in the histogram.	N/A
Median Absolute Deviation	The median absolute deviation of the occurence probability values in the histogram.	N/A
Percentile	Percentiles of the occurence probability values in the histogram.	Percentile=5 10 15 20 25 30 35 40 45 50 55 60 65 70 75 80 85 90 95;
Percentile Area	Percentiles of values in the accumulative histogram.	Percentile=5 10 15 20 25 30 35 40 45 50 55 60 65 70 75 80 85 90 95;
Quantile	Quantiles of the occurence probability values in the histogram.	Quantile=0.025 0.25 0.5 0.75 0.975;
Range	Measure the range (Max Value -Min Value) of the occurence probability values in the histogram.	N/A
Skewness	Measure the asymmetry of the occurence probability values in the histogram.	N/A

Category: shape

Variable	Description	Formula	Parameters
Compactness 1	N/A	(Volume)/(sqrt(pi)*(Surface Area)^(2/3))	N/A
Compactness 2	N/A	36*pi*(Volume^2)/((Surface Area)^3)	N/A
Convex	1. Measure the proportion of the pixels in the convex hull that are also in the region.<br>2. Refer to MATLAB "regionprops (Mask, 'Solidity')" for details	1. Compute convex value in 2D slice-by-slice. Convex=regionprops (2DMask, 'Solidity')<br>2. Compute the mean of convex value among the slices.	N/A
Convex Hull Volume	The mean volume of the 2D convex hulls that are the convex envelopes of each slice's binary mask.	N/A	N/A
Convex Hull Volume3D	3D volume of the convex hull that is the convex envelope of binary mask.	N/A	N/A
Mass	Mass caculation is only meaningful to CT images.	N/A	N/A
Max3D Diameter	Max3DDiameter= largest pairwise Euclidean	N/A	N/A

	distance between voxels on the surface of the tumor volume.		
Mean Breadth	MeanBreath=Integral of mean curvature	N/A	N/A
Number of Objects	N/A	N/A	N/A
Number of Voxel	The number of voxels treating the edge voxels differently.	N/A	Edge Voxel Fraction=0.5;
Orientation	1. Measure the angle between the x-axis and the major axis of the ellipse in 2D.<br>2. Refer to MATLAB "regionprops (Mask, 'Orientation')" for details	1. First, compute orientation value in 2D slice-by-slice. Orientation=regionprops(2D Mask, 'Orientation')<br>2. Then, compute the mean of orientation value among the slices.	N/A
Roundness	1. Measure how much the binary mask is close to circle in 2D.<br>2. Refer to MATLAB "regionprops(Mask, 'Eccentricity')" for details	1. First, compute roundness value in 2D slice-by-slice. Roundness=1-regionprops(2DMask, 'Eccentricity')<br>2. Then, compute the mean of roundness value among the slices.	N/A
Spherical Disproportion	N/A	N/A	N/A

Sphericity	N/A	N/A	N/A
Surface Area	The surface area of the binary mask.	N/A	N/A
Surface Area Density	Surface Area Density == Surface Volume Ration in Hugo's paper below.	(surface area of the binary mask)/(volume of the binary mask).	N/A
Volume	The physical volume treating the edge voxels differently.	N/A	Edge Voxel Fraction =0.5;
Voxel Size	The physical voxel size.	N/A	N/A

Category: Gray Level Cooccurence Matrix 25

Variable	Description
Auto Correlation	Auto Correlation =-333-1, -333-4, -333-7, 0-1, 0-4, 0-7, 45-1,45-4,45-7,90-1,90-4,90-7,135-1,135-4,135-7,
Cluster Prominen	Cluster Prominen= -333-1, -333-4, -333-7, 0-1, 0-4, 0-7, 45-1,45-4,45-7,90-1,90-4,90-7,135-1,135-4,135-7,
Cluster Shade	Cluster Shade= -333-1,-333-4,-333-7,0-1,0-4,0-7,45-1, 45-4, 45-7,90-1,90-4,90-7,135-1,135-4,135-7,
Cluster Tendendcy	Cluster Tendendcy=-333-1,-333-4,-333-7,0-1,0-4,0-7, 45-1, 45-4,45-7,90-1,90-4,90-7,135-1,135-4,135-7,
Contrast	Contrast=-333-1,-333-4,-333-7, 0-1, 0-4, 0-7, 45-1, 45-4, 45-7, 90-1,90-4,90-7,135-1,135-4,135-7,
Correlation	Correlation=-333-1,-333-4,-333-7, 0-1, 0-4, 0-7, 45-1, 45-4, 45-7, 90-1,90-4,90-7,135-1,135-4,135-7,
Difference Entropy	Difference Entropy=-333-1,-333-4,-333-7, 0-1, 0-4, 0-7, 45-1,

	45-4,45-7,90-1,90-4,90-7,135-1,135-4,135-7,
Dissimilarity	Dissimilarity=-333-1,-333-4,-333-7, 0-1, 0-4, 0-7, 45-1, 45-4, 45-7,90-1,90-4,90-7,135-1,135-4,135-7,
Energy	Energy=-333-1,-333-4,-333-7, 0-1, 0-4, 0-7, 45-1, 45-4, 45-7, 90-1,90-4,90-7,135-1,135-4,135-7,
Entropy	Entropy=-333-1,-333-4,-333-7, 0-1, 0-4, 0-7, 45-1, 45-4, 45-7,90-1,90-4,90-7,135-1,135-4,135-7,
Homogeneity	Homogeneity=-333-1,-333-4,-333-7, 0-1, 0-4, 0-7, 45-1, 45-4, 45-7,90-1,90-4,90-7,135-1,135-4,135-7,
Homogeneity2	Homogeneity2=-333-1,-333-4,-333-7,0-1, 0-4, 0-7, 45-1, 45-4,45-7,90-1,90-4,90-7,135-1,135-4,135-7,
InformationMeasureCorr1	InformationMeasureCorr1=-333-1,-333-4,-333-7,0-1, 0-4, 0-7,45-1,45-4,45-7,90-1,90-4,90-7,135-1,135-4,135-7,
InformationMeasureCorr2	InformationMeasureCorr2=-333-1,-333-4,-333-7,0-1, 0-4,0-7,45-1,45-4,45-7,90-1,90-4,90-7,135-1,135-4,135-7,
Inverse Diff Moment Norm	Inverse Diff Moment Norm=-333-1,-333-4,-333-7,0-1, 0-4, 0-7,45-1,45-4,45-7,90-1,90-4,90-7,135-1,135-4,135-7,
Inverse Diff Norm	Inverse Diff Norm=-333-1,-333-4,-333-7,0-1,0-4,0-7, 45-1,45-4,45-7,90-1,90-4,90-7,135-1,135-4,135-7,
Inverse Variance	Inverse Variance=-333-1,-333-4,-333-7,0-1,0-4,0-7,45-1, 45-4,45-7,90-1,90-4,90-7,135-1,135-4,135-7,
Max Probability	Max Probability=-333-1,-333-4,-333-7, 0-1, 0-4, 0-7, 45-1, 45-4, 45-7,90-1,90-4,90-7,135-1,135-4,135-7,
Sum Average	Sum Average=-333-1,-333-4,-333-7, 0-1, 0-4, 0-7, 45-1, 45-4, 45-7,90-1,90-4,90-7,135-1,135-4,135-7,
Sum Entropy	Sum Entropy=-333-1,-333-4,-333-7, 0-1, 0-4, 0-7, 45-1, 45-4,

	45-7,90-1,90-4,90-7,135-1,135-4,135-7,
Sum Variance	Sum Variance=-333-1,-333-4,-333-7, 0-1, 0-4, 0-7, 45-1, 45-4, 45-7,90-1,90-4,90-7,135-1,135-4,135-7,
Variance	Variance=-333-1,-333-4,-333-7, 0-1, 0-4, 0-7, 45-1, 45-4, 45-7,90-1,90-4,90-7,135-1,135-4,135-7,

Category: Gray Level Run Length Matrix 25

Variable	
Gray Level Non uniformity	Gray Level Non uniformity=-33,0,90
High Gray Level Run Empha	High Gray Level Run Empha=-33,0,90
Long Run Emphasis	Long Run Emphasis=-33,0,90
Long Run High Gray Level Empha	Long Run High Gray Level Empha=-33,0,90
Long Run Low Gray Level Empha	Long Run Low Gray Level Empha=-33,0,90
Low Gray Level Run Empha	Low Gray Level Run Empha=-33,0,90
Run Length Non uniformity	Run Length Non uniformity=-33,0,90
Run Percentage	Run Percentage=-33,0,90
Short Run Emphasis	Short Run Emphasis=-33,0,90
Short Run High Gray Level Empha	Short Run High Gray Level Empha=-33,0,90
Short Run Low Gray Level Empha	Short Run Low Gray Level Empha=-33,0,90
